# A Review of Ammonia-Oxidizing Archaea and Anaerobic Ammonia-Oxidizing Bacteria in the Aquaculture Pond Environment in China

**DOI:** 10.3389/fmicb.2021.775794

**Published:** 2021-11-30

**Authors:** Shimin Lu, Xingguo Liu, Chong Liu, Guofeng Cheng, Runfeng Zhou, Yayuan Li

**Affiliations:** ^1^Fishery Machinery and Instrument Research Institute, Chinese Academy of Fishery Sciences, Shanghai, China; ^2^College of Fisheries and Life Science, Shanghai Ocean University, Shanghai, China

**Keywords:** nitrogen removal, ammonia-oxidizing archaea, anammox, pond aquaculture, nitrous oxide

## Abstract

The excessive ammonia produced in pond aquaculture processes cannot be ignored. In this review, we present the distribution and diversity of ammonia-oxidizing archaea (AOA) and anaerobic ammonia-oxidizing bacteria (AnAOB) in the pond environment. Combined with environmental conditions, we analyze the advantages of AOA and AnAOB in aquaculture water treatment and discuss the current situation of pond water treatment engineering involving these microbes. AOA and AnAOB play an important role in the nitrogen removal process of aquaculture pond water, especially in seasonal low temperatures and anoxic sediment layers. Finally, we prospect the application of bioreactors to purify pond aquaculture water using AOA and AnAOB, in autotrophic nitrogen removal, which can reduce the production of greenhouse gases (such as nitrous oxide) and is conducive to the development of environmentally sustainable pond aquaculture.

## Introduction

China is the largest aquaculture country in the world, and the proportion of aquaculture in fish production increased to 73.7% in 2016 (Food and Agriculture Organization of the United Nations and Fisheries and Aquaculture Department, 2018). In 2019, pond aquaculture accounted for 48.9% of the national aquaculture output in China, among which, the output of freshwater pond and seawater pond aquaculture was 22.3 million tons and 2.5 million tons, respectively (Fisheries Bureau and Ministry of Agriculture and Rural Affairs of the People’s Republic of China, 2020). In the process of pond aquaculture, the average nitrogen deposition rate of feed protein by aquatic animals is only about 30% (Ackefors and Enell, 1994); most organic nitrogen is retained in the pond aquaculture environment. Part of the organic nitrogen is transformed to NH_4_^+^-N by ammoniation of heterotrophic bacteria and is released into the aquaculture water, resulting in the pollution of aquaculture water and stress on the healthy growth of aquatic animals (Randall and Tsui, 2002). The release of pond aquaculture water can also cause the eutrophication of the surrounding water environment. With the increase of pond aquaculture density and total yield, more and more ammonia is produced. Survey results showed that, in 2017, the ammonia emissions from aquaculture reached 22,300 tons in China, accounting for 10.31% of agricultural ammonia emissions (Ministry of Ecological Environment et al., 2020). At present, the amount of ammonia produced by aquaculture in China cannot be ignored.

Ammonia oxidation is the first step in the nitrification process, but also the rate-limiting step (Kowalchuk and Stephen, 2001). Traditionally, it was thought that ammonia oxidation mainly depends on ammonia-oxidizing bacteria (AOB). The relatively recent discovery of ammonia-oxidizing archaea (AOA) (Könneke et al., 2005) and anaerobic ammonia-oxidizing bacteria (AnAOB) (Mulder et al., 1995) not only substantially improved our understanding of the earth nitrogen cycle, but also provided new possibilities for nitrogen removal from pond aquaculture water. Compared with AOB, many new physiological characteristics appear in AOA and AnAOB. AOA have a higher affinity for ammonia, and the concentration of ammonia-saturated substrate is lower (Straka et al., 2019). AOA has very low requirements for dissolved oxygen (DO). At a DO concentration of just 0.4 μM, it can have high ammonia oxidation activity (Stahl and de la Torre, 2012). Compared with heterotrophic denitrification, low levels of organic carbon are involved in the anammox process (Mulder et al., 1995). When the water temperature is lower than 20°C, the activity of AOB decreases sharply (Hooper and Terry, 1973; Jiang and Bakken, 1999). However, AOA are not sensitive to temperature changes within a temperature range from 8 to 20°C (Stahl and de la Torre, 2012). The discovery of AOA and AnAOB provides an innovative tool for the advanced treatment of pond aquaculture water.

Here, we summarize the research status of AOA and AnAOB in aquaculture pond environments in chronological order and list the water quality characteristics of specific aquaculture ponds. Combined with the environmental conditions of the pond aquaculture, we analyze the advantages of AOA and AnAOB in aquaculture water treatment. In addition, we discuss the current state of pond water treatment application engineering, in which AOA and AnAOB are involved and prospect their application in the field of pond aquaculture. The purpose of this review is to explore the establishment of efficient pond aquaculture water treatment technology based on AOA or AnAOB.

## Distribution of Ammonia-Oxidizing Archaea and Anaerobic Ammonia-Oxidizing Bacteria in a Pond Environment

### Ammonia-Oxidizing Archaea

Although AOA were found as early as 2005 (Könneke et al., 2005), it was not until 2012 that Shimin et al. (2012) reported their existence in a freshwater aquaculture pond environment. Based on AOA *amoA*, through the construction of a clone library, Lu (2014) found that 80% of AOA in the surface sediments of grass carp pond belonged to group I.1b; the other 20% of AOA belonged to group I.1a. However, all the AOA attached to the fibrous roots of *Ipomoea aquatica* in a grass carp pond belonged to group I.1b (Lu, 2014). In 2015, Srithep et al. (2015) found that there were groups I.1a and I.1b in the sediment of shrimp ponds, and the majority of AOA sequences fell closer to group I.1a. Lu et al. (2016) found that the AOA in the 0- to 2-cm sediment layer of *Megalobrama amblycephala* ponds belonged to a *Nitrososphaera* cluster, while those in the deeper sediment layers belonged to a *Nitrosopumilus* cluster. Using the same experimental method, in 2017, it was found that AOA species affiliated to *Nitrososphaera*-like and *Nitrososphaera* clusters in three *Mandarin* fish ponds (Zhou et al., 2017). In 2019, metagenome analysis showed that *Nitrosopumilus* and *Nitrososphaera* were major AOA groups in all sediment layers of a grass carp aquaculture pond (Deng et al., 2020). Based on AOA *amoA*, in 2021, through the Illumina MiSeq platform analysis method, a phylogenetic tree revealed that most of the AOA in shrimp pond sediment were members of *Nitrosopumilus* and *Nitrososphaera* (Wei et al., 2021). Studies showed that AOA could be grouped into five major clusters: *Nitrosopumilus* (also known as group I.1a AOA), *Nitrosotalea* (also known as group I.1a-associated AOA), *Nitrososphaera* (also known as group I.1b AOA), *Nitrososphaera* sister cluster, and *Nitrosocaldus* (also known as thermophilic AOA, ThAOA) (Pester et al., 2012). Therefore, groups I.1a and I.1b AOA are so far considered to be the dominant species in the pond environment.

Perhaps subjected to photoinhibition (Lu S. et al., 2020), the abundance of AOA in pond aquaculture water is very low. However, abundant AOA are widespread in the surface sediments of aquaculture ponds (Table 1). Lu (2014) found that the abundance of AOA *amoA* gene in the surface sediments (0–5 cm depth) of grass carp ponds in Central China ranged from 4.21 (±2.00) × 10^5^ to 1.71 (±0.76) × 10^6^ copy g^–1^, about one order of magnitude higher than that of AOB. Lu et al. (2016) found that the abundance of the *amoA* gene ranged from 6.82 (±2.28) × 10^4^ to 7.79 (±3.88) × 10^5^ copies g^–1^ in a *M. amblycephala* pond at a 0- to 25-cm-deep sediment layer. Zhou et al. (2017) found that the average archaeal *amoA* gene copy number in three ponds feeding *Mandarin* fish was from 2.53 × 10^6^ to 4.13 × 10^6^ copies g^–1^ dry sediment. In 2020, it was found that the copy number of AOA *amoA* genes varied widely in shrimp sediment at different culture stages, ranging from 9.04 (± 0.32) × 10^5^ to 1.92 (± 2.2) × 10^6^ *amoA* gene copies per gram of dry sediment (Wei et al., 2021). At present, the existing research on the detection of AOA abundance in ponds was all based on the AOA *amoA* gene. Each AOA cell of groups I.1a and I.1b has one copy of the *amoA* gene (Liu, 2019), so it can be inferred that the abundance of AOA in pond sediments is 10^4^–10^6^ cell g^–1^ sediment.

**TABLE 1:** Studies on ammonia-oxidizing archaea (AOA) and anaerobic ammonia-oxidizing bacteria (AnAOB) in the pond environment.

Environment	AOA/AnAOB	Community	Abundance	Potential rates	Reference
0–5 cm deep sediment of grass carp ponds	AOA	80% AOA belonged to the *Nitrososphaera* cluster and 20% AOA belonged to the *Nitrosopumilus* cluster	4.22 ± 2.00 × 10^5^ to 1.71 ± 0.76 × 10^6^ *amoA* gene copies g^–1^ throughout the year	/	Lu, 2014
*Ipomoea aquatica* Forsk roots in grass carp ponds	AOA	100% AOA belonged to group I.1b	5.41 ± 0.25 × 10^3^–3.82 ± 0.37 × 10^4^ *amoA* gene copies g^–1^ *rhizosphere*	/	Lu, 2014
Shrimp pond sediment	AOA	More than 99.29% AOA in each sample fell within the *Nitrososphaera* group, the other AOA belonged to the *Nitrosopumilus* group	9.04 ± 0.32 × 10^5^ to 1.92 ± 2.2 × 10^6^ *amoA* gene copies g^–1^ dry sediment	/	Wei et al., 2021
0–2 cm deep sediment from a *Megalobrama amblycephala* pond	AOA	100% AOA belonged to the *Nitrososphaera* cluster	7.79 ± 3.88 × 10^5^ *amoA* gene copies g^–1^ sediment	/	Lu et al., 2016
10–15 cm and 20–25 cm deep sediment of a *Megalobrama amblycephala* pond	AOA	100% AOA belonged to the *Nitrosopumilus* cluster	6.82 ± 2.28 × 10^4^ to 1.69 ± 0.86 × 10^5^ *aomA* gene copies g^–1^ sediment	/	Lu et al., 2016
Crab pond water	AOA	97% AOA belonged to the *Nitrosopumilus* cluster; 3% AOA belonged to the *Nitrososphaera cluster*	/	/	Zhang et al., 2016
Grass carp ponds	AOA	*Nitrosopumilus* and *Nitrososphaera* AOA were detected in water and sediment	/	/	Deng et al., 2020
Shrimp pond sediment	AOA	80% AOA were related to group I.1a, 20% AOA belonged to group I.1b Thaumarchaeota	About 100 amoA gene copies ng^−1^ DNA	/	Srithep et al., 2015
0–5 cm deep sediment of grass carp ponds in spring	AnAOB	/	2.03 ± 0.92 × 10^5^ 16S rRNA gene copies g^–1^ sediment	/	Lu, 2014
0–5 cm deep sediment of grass carp ponds in summer	AnAOB	/	3.91 ± 1.92 × 10^5^ 16S rRNA gene copies g^–1^ sediment	/	Lu, 2014
0–5 cm deep sediment of grass carp ponds in autumn	AnAOB	/	4.56 ± 1.51 × 10^5^ 16S rRNA gene copies g^–1^ sediment	/	Lu, 2014
0–5 cm deep sediment of grass carp ponds in winter	AnAOB	/	1.46 ± 0.69 × 10^5^ 16S rRNA gene copies g^–1^ sediment	/	Lu, 2014
Surface sediment of grass carp ponds	AnAOB	Candidate division *Zixibacteria* (35.5–42.4%), Candidatus *Latescibacteria* (8.5–10.4%), and *Desulfuromonadales* (9.9–10.4%) (*Geobacter*-like) were the dominant AnAOB	/	/	Deng et al., 2020
Freshwater aquaculture pond	AnAOB	Including Candidatus *Brocadia*, Candidatus *Kuenenia*, and Candidatus *Anammoxoglobus*, with Candidatus *Brocadia* being the dominant AnAOB genus	5.6 × 10^4^ to 2.1 × 10^5^ *hzs* gene copies g^–1^ sediment	3.7–19.4 nmol N_2_ g^–1^ sediment day^–1^; Contribution to sediment N_2_ ranged from 1.2 to 15.3%	Shen et al., 2016
Shrimp aquaculture ponds	AnAOB	The phylogenetic tree of AnAOB based on *hzo* gene sequences showed relatedness to *Candidatus Kuenenia* and *Candidatus Scalindua* genera	10^6^ to 10^7^ *hzo* gene copies g^–1^ sediment	/	Nair et al., 2020
A semi-intensive shrimp pond	AnAOB	*Ca.* “*Kuenenia*”-like gene fragments were the major component of AnAOB	/	0.7 nmol N_2_ cm^–3^ h^–1^	Amano et al., 2011
Tropical aquaculture settlement ponds	AnAOB	/	/	0–7.07 nmol N cm^–3^ h^–1^	Castine et al., 2012

*“/” indicates no relevant data.*

Perhaps because of the difficulty in culturing AOA in the laboratory, there are few reports of AOA strains or high abundance cultures originating from fishponds. The research studies on the physical and chemical properties of AOA are almost all based on the statistical analysis of AOA and environmental factor data. For instance, it was found that both the abundance and diversity of AOA were significantly negative to the concentration of ammonium in interstitial water (Zhou et al., 2017). Correlation analyses indicated a significant correlation between the abundance of AOA and total nitrogen (TN) and arylsulfatase, and AOA diversity was significantly correlated with β-glucosidase (Dai et al., 2018). The conclusion was that AOA communities in the surface sediments of aquaculture ponds were regulated by organic matter. The AOA community has a close relationship with total organic carbon (TOC), pH, total phosphorus, nitrate reductase, urease, acid phosphatase, and β-glucosidase (Wei et al., 2021).

### Anaerobic Ammonia-Oxidizing Bacteria

Mulder et al. (1995) confirmed that anammox was driven by microorganisms by using isotope tracer technology. In the reaction process, AnAOB uses one molecule of ammonia and one molecule of nitrite to produce one molecule of nitrogen gas, and with no need to supplement an organic carbon source in the process. Later, it was found that anammox was responsible for 24–67% of nitrogen loss in marine sediments (Thamdrup and Dalsgaard, 2002). Moreover, the anammox phenomenon existed in many other environments, such as lakes (Schubert et al., 2006), rivers (Zhang et al., 2007), and soil (Zhu et al., 2011). In recent years, technology using anammox in wastewater treatment has been widely applied around the world (Lackner et al., 2014; Weralupitiya et al., 2021).

The anammox phenomenon is also associated with the pond aquaculture environment (Table 1). In 2011, it was reported that the rate of potential anammox activity was 0.7 nmol N_2_ cm^–3^ h^–1^, and corresponded to at most 2% of the denitrification. *Kuenenia*-like AnAOB were the major component recovered from shrimp pond sediment (Amano et al., 2011). Lu (2014) found AnAOB accompanied by AOA in the surface sediments of freshwater aquaculture ponds. The abundance of AnAOB 16S rRNA ranged from 1.46 (± 0.69) × 10^5^ to 4.56 (± 1.51) × 10^5^ copies g^–1^ sediment throughout the whole year, and the highest abundance occurred in the autumn when the water temperature was 18°C (Lu, 2014). Shen et al. (2016) found that the abundance of the AaAOB *hzs* gene ranged from 5.6 × 10^4^ to 2.1 × 10^5^ copies g^–1^ sediment in different freshwater aquaculture ponds, and this process contributed to 1.2-15.3% of sediment N_2_ production. In the same year, both 16S rRNA and *hzo* gene diversity analyses indicated that the major AnAOB in the sediments of marine aquaculture zones were *Scalindua*-related species, although *Kuenenia*-like AnAOB could also be detected from one of the four selected sampling sites (Li and Gu, 2016). In 2018, AnAOB were stimulated with the supplementation of bicarbonate in shrimp aquaculture sediment. The enriched AnAOB achieved a maximum nitrogen removal efficiency rate of 1.00 ± 0.02 kg-N m^–3^ day^–1^ (Luong Van et al., 2018). Nair et al. (2020) found that the abundance of the AnAOB *hzo* gene ranged from 10^6^ to 10^7^ copies per gram of sediment, and the concentration of ammonia, nitrite, redox potential, and the total organic carbon showed a strong positive correlation with the abundance of AnAOB in zero water exchange aquaculture ponds. There is no doubt that Anammox is ubiquitous in pond sediments and plays an important role in the process of ammonia oxidation and denitrification. The predominant AnAOB species and its driving factors in the pond environment are still uncertain.

## Opportunity for Nitrogen Removal by Ammonia-Oxidizing Archaea and Anaerobic Ammonia-Oxidizing Bacteria in Aquaculture Ponds

As mentioned above, AOA and AnAOB are widespread in the aquaculture pond environment, which means the nutrient elements of fishponds are suitable for the growth of AOA and AnAOB. Here, combined with the characteristics of the aquaculture pond, we analyze the feasibility of pond aquaculture water purification using AOA and AnAOB, from the perspective of DO, temperature, organic matter, ammonia, and pH.

### Temperature

Pond aquaculture is widely distributed in China and Southeast Asian countries. Generally, fish, shrimp, or crab fry start feeding when the water temperature rises every spring. After the summer to the end of autumn or winter, when breeding conditions are unsuitable for feeding due to low water temperature, drainage is started and nets are used to capture activity. In one aquaculture year, the temperature of the pond water varies widely. For example, in Central China, in the largest pond aquaculture area in China, the water temperature ranges from 5 to 28°C throughout the year and is only 4–18°C in autumn and winter (Lu et al., 2015). AOB activities could be severely inhibited under low temperatures (<15°C) (Zhang et al., 2020). AOA can compensate for the deficiency of AOB ammonia oxidation activity at low temperatures. AOA is more resistant to low temperature, and it is reported that they are not sensitive to temperature changes within 8–20°C (Stahl and de la Torre, 2012). In freshwater pond sediment, it was found that the maximum abundance of AOA occurred in winter, when the water temperature was 4°C, while the maximum abundance of AOB occurred in the autumn, when the water temperature was 18°C (Lu et al., 2015), indicating that AOA makes a greater contribution to ammonia oxidation in the pond environment at low temperatures. Similar phenomena occurred in some biofilm reactors. It was evaluated that up to 94.9% of the overall ammonia oxidation could be attributed to AOA at 10°C, 48.2% of that was undertaken by AOA at 35°C (Lin et al., 2020).

As with AOA, AnAOB also can adapt to a wide range of temperatures. It was reported that anammox can take place at temperatures from 6 to 43°C; the reaction rate drops rapidly at temperatures lower than 15°C or higher than 40°C (Ma et al., 2016). Although few studies have reported that AnAOB can adapt to a low temperature pond environment, it was documented in some anammox reactors. For instance, in an up-flow anaerobic sludge blanket, a high nitrogen removal rate of 2.28 kg N m^–3^ day^–1^ was achieved at a temperature of 16°C (Ma et al., 2013). In a gel carrier with entrapped AnAOB, anammox activity still occurred at 6.3°C. The nitrogen conversion rates at 22 and 6.3°C were 2.8 and 0.36 kg N m^–3^ day^–1^, respectively (Isaka et al., 2008). This discovery has developed our understanding of AOA and AnAOB in the nitrogen cycling of pond aquaculture at low seasonal temperatures.

### Dissolved Oxygen

The aquaculture animals in fishponds need high concentrations of DO, and on sunny days, in surface aquaculture water, the DO is often in a supersaturated state, whereas the subsurface sediment of the pond is anoxic. It was reported that the depth of the DO detection limit was 500 μm in freshwater pond surface sediment, and the DO concentrations ranged from 0 to 48.01 μmol L^–1^ (Lu et al., 2016). In contrast to AOB, AOA is more suitable for low DO in pond surface sediments. AOA often thrive at DO levels of ca. 3 μM and can achieve higher ammonia oxidation under oxygen-limited conditions (Stahl and de la Torre, 2012). AOA has an affinity for oxygen that is more typical of aerobic microorganisms (∼4 μM) and is unable to grow anaerobically under the culture conditions so far evaluated (Stahl and de la Torre, 2012). For example, the AOA enrichment of *Nitrososphaera viennensis* had kinetic km, O_2_ ≈ 2.9 μM (Straka et al., 2019); however, the oxygen half-saturation constant of AOB was 21.4–56.4 μM in continuous stirred tank reactors (Guisasola et al., 2005; Manser et al., 2005; Daebel et al., 2007; Jubany et al., 2008). AOA could outcompete AOB and occupy the corresponding niche under anoxic sediment.

Unlike AOA and AOB, AnAOB is a type of anaerobic ammonia-oxidizing microorganism, whose activity could be inhibited by the presence of oxygen (Liu et al., 2016). AnAOB needs substrate NO_2_^–^-N, which could only be provided by AOA or AOB in a pond environment. Therefore, in the surface sediment, AnAOB always exists with AOA and AOB in the autotrophic nitrogen removal process. For example, AOA *Nitrosopumilus* sp., *Nitrososphaera* sp., AOB *Nitrosomonas* sp., and AnAOB Candidatus *Kuenenia* sp. were found to be the dominant ammonia-oxidizing communities in zero water exchange shrimp ponds (Nair et al., 2019, 2020). Throughout the year, there were AnAOB, AOA, and AOB in the surface sediments of the grass carp pond. Moreover, there was a significant positive correlation between the abundance of AOA *amoA* and AnAOB 16S rRNA genes in the winter, which indicates that there is a synergistic effect between AOA and AnAOB in pond sediments in winter (Lu, 2014).

**FIGURE 1:**
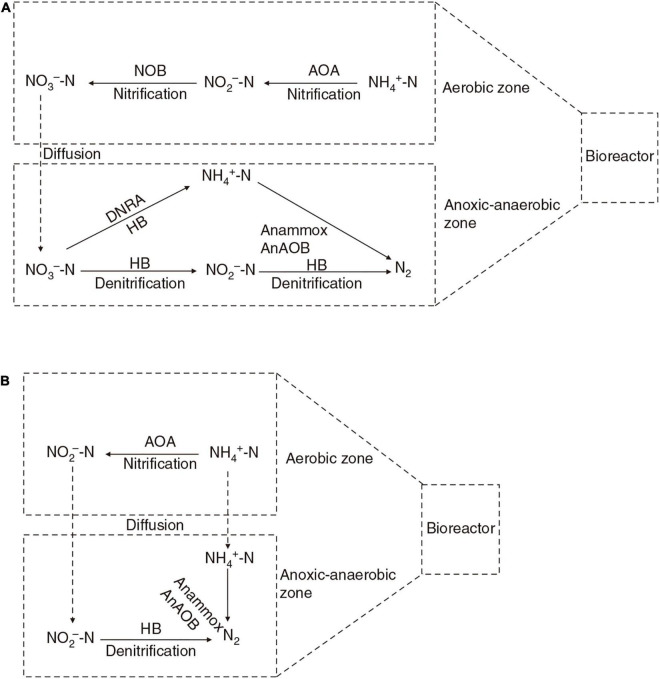
Cooperative working mechanism of AOA and AnAOB in bioreactor. AOA, ammonia-oxidizing archaea; NOB, nitrite-oxidizing bacteria; AnAOB, anaerobic ammonia-oxidizing bacteria; HB, heterotrophic denitrifying bacteria; DNRA, dissimilatory nitrate reduction to ammonium process. Solid arrows represent different nitrogen cycling processes, and the dashed arrow represents diffusion in bioreactor. AnAOB use nitrite and ammonia generated by heterotrophic denitrification and dissimilatory nitrate reduction process in the anoxic–anaerobic zone of biofilm **(A)**, where the nitrate concentration is high but ammonia and nitrite are scarce. Alternatively, AnAOB can directly use nitrite produced by AOA and ammonia generated by heterotrophic ammonification, where nitrite-oxidizing bacteria are inhibited **(B)**.

The AnAOB activity could be inhibited by the presence of oxygen (Liu et al., 2016); so far, no anammox activity has been reported in pond aquaculture water. However, AnAOB can be detected in aerated systems, such as nitrogen-removing moving bed biofilters (van Kessel et al., 2010; Persson et al., 2014), rotating biological contactor and biofilters (Egli et al., 2003), and the simultaneous partial nitrification, anammox, and denitrification (SNAD) bioreactor (Lu J. et al., 2020). They probably inhabit the oxygen-depleted zones of aerated biofilters (Mulder et al., 1995), where an anoxic environment is created by AOA, AOB, and heterotrophic bacteria. AnAOB use nitrite and ammonia generated by heterotrophic denitrification and the dissimilatory nitrate reduction process (Hao et al., 2021) (Figure 1A), where nitrate concentration is high but ammonia and nitrite are scarce. Alternatively, AnAOB can use nitrite produced by AOA and AOB and ammonia generated by heterotrophic ammonification, in what is called the one-stage nitritation/anammox process (Li et al., 2018) (Figure 1B).

Because the anammox process takes ammonia as the electron donor and nitrite as the electron acceptor to directly generate nitrogen gas, there is no need to oxidize nitrite into nitrate, only oxygen is required in the ammonia oxidation process. Compared with the traditional nitrification-denitrification water treatment process, the total energy for aeration can be reduced by 60% (Moomen and Ahmed, 2018). Therefore, bioreactors based on AOA and AnAOB are expected to be used in the treatment of pond aquaculture water in the future.

### Ammonia

In the pond aquaculture environment, the ammonia concentrations between aquaculture water and pore water of surface sediment are quite different. For example, the survey results of 10 grass carp ponds showed that the ammonia concentration in sediment pore water is 3.30 ± 0.88 mM L^–1^, while the ammonia concentration in aquaculture water is only 0.16 ± 0.13 mM L^–1^ (Lu et al., 2015). From the perspective of tolerance to ammonia concentration, some AOA strains may not adapt to the high ammonia concentration level in sediment pore water. For example, a study on AOA isolated from a recirculating aquaculture system (RAS) showed that the ammonia concentration of 0.25–1 mM L^–1^ is suitable for the growth of AOA. When the concentration of ammonia reaches 2 mM L^–1^, the growth of AOA appears to be inhibited (Sauder et al., 2018). In enrichment cultures, AOA-DW and AOA-AC5 showed the highest growth at the ammonia concentration of 1 and 2 mM L^–1^, respectively (French et al., 2012). However, some AOA strains or cultures can endure higher ammonia concentrations, such as the high tolerances to NH_4_^+^ observed for *N*. *viennensis* (15 mM L^–1^) (Tourna et al., 2011), Ca. *Nitrosoarchaeum koreensis* (10 mM L^–1^), and a strain in the AOA AC2 enrichment culture (5 mM L^–1^) (French et al., 2012). It was found that group I.1b AOA species could tolerate very high ammonium concentrations (Jung et al., 2021).

As mentioned above, the AOA of the pond aquaculture environment mainly belong to group I.1a and group I.1 b. The latest research also showed that the AOA of group I.1a and group I.1b had a wide range of cellular ammonia affinities (Jung et al., 2021). Group I.1a AOA displayed a high substrate affinity, and the ammonia apparent-half-saturation concentration Km(app), which ranged from ∼2.2 to 24.8 nM, can adapt to oligotrophic conditions, while several group I.1b species possess a wide range of affinities for NH_3_ [Km(app) = ∼0.14 to 31.5 μM] (Jung et al., 2021). Compared with AOB, AOA may not have the advantage of using high-concentration ammonia. However, the AOA strains showed a higher affinity for ammonia than published AOB measurements (>20 μM) (Straka et al., 2019). Throughout the year, the concentrations of pond aquaculture water ammonia are less than 0.21 mM L^–1^ most of the time (Table 2). Therefore, AOA has greater advantages than AOB in the advanced treatment of aquaculture water with a low ammonia nitrogen concentration. Because pond aquaculture facilities are relatively simple, few reports exist on using bioreactors to treat pond aquaculture water based on AOA. However, it was well documented in a limit water exchange recirculating freshwater aquarium using a down-flow hanging sponge and up-flow sludge blanket system to remove nitrogen, in which only AOA was detected, and no AOB existed (Adlin et al., 2017).

**TABLE 2:** Characteristics of pond aquaculture water and pore water of sediment.

Pond	NH_4_^+^-N (mM L^–1^)	NO_2_^–^-N (μM L^–1^)	NO_3_^–^-N (μM L^–1^)	COD (mg L^–1^)	Reference
Surface water of grass carp ponds	0.07–0.18	1.43–17.14	15.00–59.29	/	Qin, 2016
Bottom water of grass carp ponds	0.07–0.19	1.43–17.14	17.86–60.71	/	Qin, 2016
Surface water of grass carp ponds in spring	0.090.02	5.002.14	9.293.57	/	Lu et al., 2015
Surface water of grass carp ponds in summer	0.20.07	24.2925.71	9.292.86	/	Lu et al., 2015
Surface water of grass carp ponds in autumn	0.160.13	9.286.43	40.7118.57	/	Lu et al., 2015
Surface water of grass carp ponds in winter	0.070.06	5.002.14	40.7123.57	/	Lu et al., 2015
0.3 m below the surface water of shrimp pond	0.005 ± 0.002–0.008 ± 0.002	2.14 ± 0.64–10.07 ± 2.57	29.57 ± 5.93–55.43 ± 15.14	3.84 ± 0.24–4.62 ± 0.24	Li et al., 2021
At 0.6 m depth water of a *Penaeus vannamei* pond	0.004–0.07	11.43–55.71	8.57–32.14	/	Li et al., 2019
Freshwater aquaculture pond	0.04–0.07	/	/	2.9–4.0	Li and Li, 2009
An intensive pond aquaculture system	0.04 ± 0.004–0.13 ± 0.003	6.43 ± 0.00–43.57 ± 1.43	/	/	Bao et al., 2018
*Mandarin* fish ponds	0.01–0.14	/	3.57 ± 1.43–582.86 ± 11.43	8.32 ± 0.23–15.64 ± 1.20	Zhou et al., 2017
Shrimp ponds	0.03 ± 0.04–0.05 ± 0.07	−24.2942.14	3.57 ± 5.00–43.57 ± 57.14	53.5 ± 25.8–99.9 ± 23.9	Lin et al., 2010
Pore water of grass carp pond sediment	0.820.17	2.140.71	142.1427.86	/	Deng et al., 2020
Pore water of freshwater aquaculture pond sediment	1.2–3.4	2.14–0.004	11.43–25.71	/	Shen et al., 2016
Pore water of grass carp pond sediment in spring	1.690.92	0.710.00	8.572.86	/	Lu et al., 2015
Pore water of grass carp pond sediment in summer	3.300.88	7.865.71	9.292.86	/	Lu et al., 2015
Pore water of grass carp pond sediment in autumn	1.090.41	0.710.00	10.712.14	/	Lu et al., 2015
Pore water of grass carp pond sediment in winter	1.440.50	1.430.00	12.862.86	/	Lu et al., 2015
Pond sediment	4.960.11	450.0099.29	1,063.5770.71	/	Wei et al., 2021
*Mandarin* fish pond sediment	0.15 ± 0.01–0.28 ± 0.002	/	2.86 ± 0.00–35.71 ± 3.57	/	Zhou et al., 2017

*“/” indicates no relevant data.*

Unlike AOA, AnAOB prefer to live in an environment with high concentrations of ammonium, and it was reported that AnAOB was inhibited only when ammonium concentrations are above 71.43 mM L^–1^ (Jin et al., 2012). As shown in Table 2, the concentration of pore water ammonia is never higher than 7.14 mM L^–1^, indicating that AnAOB could not be inhibited by the sediment ammonia. AnAOB can also grow well in an environment with low ammonia concentration, this phenomenon is apparent in a marine environment. For example, the ammonium concentration is no more than 0.1 mM L^–1^ in the deep water of the black sea, where the abundance of AnAOB ranged from 300 to 3,000 cells mL^–1^ in the suboxic zone, and consume more than 40% of the fixed nitrogen (Kuypers et al., 2003). As shown in Table 2, the concentration of ammonia in pond aquaculture water is generally maintained above 0.07 mM L^–1^, indicating that the ammonia concentrations of the pond environment are suitable for AnAOB growth. As discussed above, a bioreactor may need to be established, when removing aquaculture water nitrogen using AnAOB and AOA.

### Organic Matter

The AOB is a strictly chemoautotrophic microorganism and cannot use organic matter (Jin et al., 2012). However, a genomic bioinformatic analysis showed that AOA has the genetic potential for mixed trophic metabolism (Walker et al., 2010). AOA genomes encode five carbohydrate-active enzyme families: glycoside hydrolases, glycosyl transferases, carbohydrate esterases, carbohydrate-binding modules, and auxiliary activities (Liu, 2019). Moreover, it was found that the AOA genome encodes organic transport protein families, such as the divalent anion Na^+^ symporter family, which has the ability to transport extracellular succinate, malate, aspartic acid, α-ketoglutarate, etc. (Liu, 2019). Isolated AOA strains also showed mixed trophic phenomenon that can utilize some organic carbon compounds, such as α-ketoglutarate (Qin et al., 2014), cyanate (Palatinszky et al., 2015), urea (Tolar et al., 2016), and pyruvic acid (Tourna et al., 2011), although the association between these characterizations and the AOA genomic features has not been demonstrated. In addition, the AOA strain *Nitrososphaera gargensis* could degrade sulfonamide antibiotics through deamination, hydroxylation, and nitrification (Zhou et al., 2019). Perhaps, its ability to degrade and utilize organic compounds leads to its widespread presence in the pond sediment.

At present, residual bait and feces are directly left at the bottom of the aquaculture pond, and the aquaculture water is in close contact with the sediment. In most cases, the nitrate in the aquaculture water body will not accumulate in large quantities (Table 2). However, the organic matter of aquaculture water is often insufficient to support heterotrophic denitrification in the complete removal of nitrate from the water, without the sediment. It is reported that in the circulating water culture system of *Paralichthys olivaceus*, the concentration of nitrate even exceeds 35.71 mM L^–1^ (Honda et al., 1993). AnAOB are chemoautotrophic microorganisms with CO_2_ as the main carbon source (Kuenen, 2008). In the anammox process, ammonia can be the electron donor, and little organic carbon is required. Autotrophic anammox can make up for the lack of heterotrophic denitrification function. It is generally thought that abundant organic matter has adverse effects on AnAOB (van de Graaf et al., 1996; Tang et al., 2010). It was reported that anammox activity can be inhibited, when the chemical oxygen consumption (COD) of pig manure effluent is >237 mg L^–1^ (Molinuevo et al., 2009). As shown in Table 2, the COD of pond aquaculture water is less than 100 mg L^–1^. Hence, it can be inferred that the concentration of organic matter in pond aquaculture water will not inhibit AnAOB. Although at present, there is no report about suspended or attached AnAOB in pond aquaculture water, as mentioned above, AnAOB that could effectively be integrated in RAS biofilter systems had to be found (van Kessel et al., 2010). The organic matter composition of pond water is similar to that of RAS aquaculture water; both mainly originate from feces and residual bait. Therefore, it is very possible to purify pond aquaculture water by biofilter systems with attached AnAOB, coupling AOA and AOB, for autotrophic nitrogen removal, in the absence of sediment.

### pH

The pH affects the ratio of ammonium ions (NH_4_^+^) to ammonia molecules (NH_3_) and then affects the ammonia oxidation function of AOA (He et al., 2012). In the breeding season, the ideal pH range of a healthy freshwater pond within 24 h is 6.5–9.0 (Wurts and Durborow, 1992). At present, most studies have shown that the best pH for the growth of group I.1a or group I.1b AOA is close to 7.0. For example, AOA strain JG1 belonging to group I.1b shows an optimal pH for ammonia oxidation of 6.5–7.0, the ammonia oxidation rate decreased below pH 6, and no sign of ammonia oxidation was observed at pH 5.5 or 8.0 (Kim et al., 2012). Three AOA cultures belonging to thaumarchaeal group I.1a grow at almost the same rate over a wide pH range of 6.5–9.0, with the highest growth rate occurring at pH 7 to 7.5 (French et al., 2012). On sunny days, the pH of surface pond water is not suitable for AOA growth. However, research has shown that the pH is very different between the surface and bottom water of aquaculture ponds in sunny weather. The pH of the bottom pond water is still close to 7.0 when the pH of the surface water approaches 9.0 (Deng et al., 2020). The pond bottom water can provide stable pH for the growth of AOA.

It has been reported that AnAOB have a physiological pH of 6.5 to 9.0 (Oshiki et al., 2016). The tolerance of AnAOB to pH is dependent on the species. For example, the optimal pH for Ca. *Brocadia sinica* was 7.0–8.8, while Ca. *Brocadia* sp. 40 had an optimal pH of 6.8–7.5 (Oshiki et al., 2016). The metabolic activity of AnAOB can be suppressed when the pH value is too high. It was found that the anammox bioreactor became destabilized when the effluent pH increased to 8.7–9.1, accompanied by the free ammonia concentration of 4.57–5.21 mM L^–1^ (Tang et al., 2009). In anammox reactors, a high pH should be avoided, a pH near neutral is the best option (Ma et al., 2016). As mentioned above, the pH of the bottom pond water is close to 7.0, which can meet the needs of both AOA and AnAOB.

### Nitrous Oxide

Nitrous oxide (N_2_O) is a strong greenhouse gas, which has a Ca. 300-times-higher warming impact than CO_2_, responsible for 5–7% of the observed greenhouse effect (Rahn, 1997; Fux and Siegrist, 2004; Santoro et al., 2011). Tropospheric concentrations of N_2_O are rising at a rate of ∼0.25% per year (Santoro et al., 2011). In biological wastewater treatment, microbial processes such as autotrophic nitrification and heterotrophic denitrification have been identified as major sources (Pw et al., 2012). It was documented that NO is an intermediate metabolite of AnAOB, but it cannot be further oxidized to N_2_O, and AnAOB cannot produce N_2_O (Fux and Siegrist, 2004; Kartal et al., 2010). AOB can produce N_2_O during the oxidation of ammonia (NH_3_) to nitrite (NO_2_^–^) and reduction of NO_2_^–^ to N_2_O in a process frequently termed “nitrifier-denitrification” (Ostrom et al., 2000). N_2_O can also be produced by AOA; however, a study has shown that the total amount of N_2_O accumulated by strains of group I.1a was far lower than that produced by AOB of *Nitrosomonas europaea*. The N_2_O production of AOA strains was only 18% of that produced by *N. europaea*, when generating the same amount of NO_2_^–^ (Jung et al., 2011). As mentioned above, AOA in the pond aquaculture environment mainly belongs to group I.1a and group I.1b, and AOB mainly belongs to *N. europaea* (Lu et al., 2019). Therefore, AOA replaces AOB for ammonia oxidation, and AnAOB replaces heterotrophic denitrification, to some extent, which can reduce the production of greenhouse gases and is conducive to the development of environmentally sustainable pond aquaculture.

## Current Application and Prospects of Ammonia-Oxidizing Archaea and Anaerobic Ammonia-Oxidizing Bacteria in Pond Aquaculture

### Applications

To develop eco-friendly pond aquaculture, the impact of aquaculture tailwater on the surrounding water environment needs to be minimized. Now, many governments have formulated strict standards of aquaculture wastewater discharge. For example, in China, the government requires that the TN of freshwater pond tailwater should not exceed 0.21 mM L^–1^ (Requirement for Water Discharge from Freshwater Aquaculture Pond, SC/T9101-2007). Specific ecological engineering measures are taken for purifying pond aquaculture water, which includes *in situ* water treatment facilities, such as *in situ* biological floating beds (Lu, 2014) and ecological slopes (Liu X. et al., 2021), and ectopic water treatment facilities, such as artificial subsurface flow wetland (Liu X. et al., 2021) and settlement ponds (Castine et al., 2012). These engineering measures during nitrogen removal processes more or less involve AOA or AnAOB. For example, a biological floating bed is usually set up on the surface of the pond water to purify aquaculture water; the developed rhizosphere of the aquatic plants can create a good habitat for the growth of AOA and AOB and rely on their attachment to accelerate the nitrogen transformation of the aquaculture environment. The abundance of AOA in the floating bed rhizosphere of water spinach in grass carp ponds was up to ∼10^5^ cell g^–1^ rhizosphere (Lu, 2014); however, at the same time, the abundance of AOA in the surrounding aquaculture water was below the detection limit (Lu, 2014). One of the purposes of a pond ecological slope is to promote the transformation of ammonia to nitrite using an attachment biofilm. At present, there is a lack of understanding of the water purification mechanism of the pond ecological slope, and the abundance and species of AOA on the pond ecological slope are still not clear. However, a large number of AOA and AOB are found in the biofilms of stream pebbles, which is similar to the pond environment (Merbt et al., 2017).

Perhaps, considering the cost of water treatment, a large-scale bioreactor has not been used to purify pond aquaculture water. However, laboratory-scale SNAD bioreactors and sequencing batch reactors (Lyles et al., 2008) have been studied for the treatment of aquaculture wastewater and have achieved a good result. Research has shown a bioreactor start-up for 180 days without adding extra organic carbon. After a 60-day operation, the bioreactor reached a stable stage with the average concentration of ammonia/nitrate/nitrite/COD in the effluent of 0.26/0.75/0.47/0.27 mg L^–1^ (Lu J. et al., 2020). Because they do not need extra organic carbon and are energy-saving, SNAD bioreactors have good prospects.

In the past decade, subsurface flow constructed wetlands (SSFCWs) have been used to treat the particulate and dissolved fraction of aquaculture wastewater from land-based fish farms (Snow et al., 2012). Recently, it was reported that AnAOB existed in the SSFCWs and that 77.72% of the AnAOB belong to Ca. *Brocadia* (Liu X.-G. et al., 2021). Before the pond aquaculture water enters the artificial subsurface flow wetland, it is often pretreated by sedimentation (Liu X. et al., 2021). It was reported that heterotrophic bacteria denitrification was the main driver of N_2_ production, with anammox detected in two of four settlement ponds (Castine et al., 2012). Perhaps, too much organic carbon could inhibit the anammox of the settlement ponds. Heterotrophic bacteria can rapidly grow and compete for nitrite and living space with anammox under high organic loading conditions (Chamchoi et al., 2008; Jenni et al., 2014).

### Prospects

**FIGURE 2:**
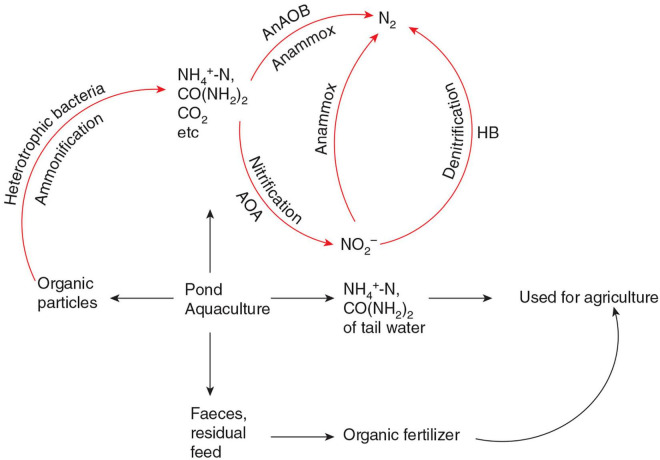
Schematic diagram of a pond aquaculture water treatment process to be established in the future. AOA, ammonia-oxidizing archaea; AnAOB, anaerobic ammonia-oxidizing bacteria; HB, heterotrophic denitrifying bacteria. Red arrows represent different nitrogen cycling processes in the pond aquaculture water treatment process. Black arrows represent the flow direction of pond organic matter rich in nitrogen. Feces and residual feed are further processed and become agricultural organic fertilizer. Part of pond aquaculture tail water rich in NH_4_^+^ and CO(NH_2_)_2_ can be directly used for agricultural irrigation. Most NH_4_^+^ and CO(NH_2_)_2_ are directly generated by aquaculture animals or arise from ammonification by heterotrophic bacteria mainly removed via nitrification and anammox by AOA and AnAOB.

Nitrogen pollution is one of the limiting factors of the sustainable and healthy development of the pond aquaculture model. The sediment is the main pollution source of pond aquaculture water, it should be removed from the aquaculture environment as far as possible by sedimentation, filtration, or other methods. Because the main components of pond aquaculture sediments are the feces and residual feed, which are rich in nitrogen, phosphorus, and other elements, the separated sediment can be used as raw material for agricultural organic fertilizer. For the micro-organic particles and dissolved organic matter that cannot be removed by physical methods in pond aquaculture water, it is recommended that they are removed by aquaculture water bioreactors, which are rich in AOA, AOB, and AnAOB. The bioreactor physical and chemical factors required by AOA and AnAOB, are easier to achieve compared with wetland, ecological floating bed, etc. In the bioreactors, the organic nitrogen of micro-organic particles and dissolved organic matter were degraded into ammonia and urea by heterotrophic bacteria. After ammonia oxidation and anaerobic ammonia oxidation, the organic nitrogen is finally formed into N_2_. Because the nitrate of aquaculture water usually accumulates to a very high level without sediment (Honda et al., 1993), there is not enough organic carbon in the water for heterotrophic denitrification. AnAOB can carry out denitrification without an additional organic carbon source, which will save a lot of water treatment costs. The bioreactors can be moving bed biofilters, SNAD bioreactors, etc. (Lu J. et al., 2020). This bioreactor can greatly reduce N_2_O emissions during pond aquaculture because the nitrogen removal process is mainly carried out by AOA and AnAOB. A schematic diagram of a pond aquaculture water treatment process to be established in the future is shown in Figure 2.

### Challenges

As mentioned above, AOA and AnAOB have many advantages in pond aquaculture water treatment. For example, they can maintain high biological activity under low temperatures. In the process of nitrogen removal, there is no need for additional organic carbon. AOA and AnAOB can perform adherent growth, which is suitable for the establishment of a high-efficiency bioreactor. However, the following difficulties still need to be overcome in production practice:

(1) AOA and AnAOB are autotrophic microorganisms with a slow growth rate. The growth rate of AOA enrichments is approximately 0.23–0.36 day^–1^, and the max growth rate of AnAOB is only 0.16 day^–1^ (Straka et al., 2019). The typical doubling times are approximately 15–30 days (Joss et al., 2009; Li et al., 2018). Generally, the start-up time of an AnAOB bioreactor exceeds 200 days (Ge et al., 2008; Yu et al., 2013; Adams et al., 2020), and the start-up time even exceeds a pond breeding cycle. (2) The discharge time of pond aquaculture tailwater is relatively concentrated, and the one-time drainage is large, which may require large-scale water treatment reactors, but these facilities are idle for most of the time, and the enriched AOA or AnAOB biofilm needs long-term maintenance. Compared with RAS aquaculture, the density of pond aquaculture is low. For example, in China, the average density of freshwater pond aquaculture is 8,400 kg km^2^ when captured (Fisheries Bureau and Ministry of Agriculture and Rural Affairs of the People’s Republic of China, 2020). In the first and middle stages of one aquaculture year, the aquaculture biomass is very small, the amount of feeding is low, and the aquaculture water can be completely treated by the self-purification ability of the environment, with no need for further treatment by peripheral equipment. Only in the late stage and at the end of one aquaculture year, especially when capturing, it is necessary to treat the discharge pond water. (3) It is difficult to screen and culture AOA and AnAOB, which are suitable for low-temperature aquaculture water. The pond aquaculture model is closely associated with temperature changes. Fish cultured at normal temperatures, such as grass carp and carp, are often captured when water temperatures are low. Although previous studies have shown that, some AOA and AnAOB can adapt to low-temperature environments. However, it is difficult to obtain AOA and AnAOB under normal temperature conditions. Perhaps, it is necessary to create low-temperature conditions for the growth of AOA and AnAOB over a long time, which is a great expense. How to obtain AOA and AnAOB enrichment cultures that are suitable for low-temperature conditions at a low cost is a major problem to be considered in the future.

## Conclusion

Ammonia-oxidizing archaea and AnAOB exist widely in aquaculture pond sediment and play important roles in nitrogen removal processes, especially in low seasonal temperatures and anoxic sediment layers. Because AnAOB uses ammonia as the electron donor and little organic carbon source is required, anammox could compensate for the lack of heterotrophic denitrification function. The concentration of aquaculture water ammonia, organic matter, and pH fit the growth of AOA and AnAOB. However, AOA and AnAOB cannot grow well naturally in aquaculture water. It is very possible to purify the pond aquaculture water by bioreactors based on AOA and AnAOB for autotrophic nitrogen removal, which can reduce the production of greenhouse gas and is conducive to the development of environmentally sustainable pond aquaculture.

## Author Contributions

SL and XL contributed to conception and design of the study. CL wrote the first draft of the manuscript. RZ and YL wrote sections of the manuscript. All authors contributed to manuscript revision, read, and approved the submitted version.

## Conflict of Interest

The authors declare that the research was conducted in the absence of any commercial or financial relationships that could be construed as a potential conflict of interest.

## Publisher’s Note

All claims expressed in this article are solely those of the authors and do not necessarily represent those of their affiliated organizations, or those of the publisher, the editors and the reviewers. Any product that may be evaluated in this article, or claim that may be made by its manufacturer, is not guaranteed or endorsed by the publisher.
